# Protective, Antioxidant and Antiproliferative Activity of Grapefruit IntegroPectin on SH-SY5Y Cells

**DOI:** 10.3390/ijms22179368

**Published:** 2021-08-29

**Authors:** Domenico Nuzzo, Miriana Scordino, Antonino Scurria, Costanza Giardina, Francesco Giordano, Francesco Meneguzzo, Giuseppa Mudò, Mario Pagliaro, Pasquale Picone, Alessandro Attanzio, Stefania Raimondo, Rosaria Ciriminna, Valentina Di Liberto

**Affiliations:** 1 Istituto per la Ricerca e l’Innovazione Biomedica, CNR, via U. La Malfa 153, 90146 Palermo, Italy; domenico.nuzzo@irib.cnr.it (D.N.); pasquale.picone@irib.cnr.it (P.P.); 2 Dipartimento di Biomedicina, Neuroscienze e Diagnostica Avanzata, Università di Palermo, Corso Tukory 129, 90134 Palermo, Italy; miriana.scordino@you.unipa.it (M.S.); costanza.giardina@unipa.it (C.G.); giuseppa.mudo@unipa.it (G.M.); 3 Istituto per lo Studio dei Materiali Nanostrutturati, CNR, via U. La Malfa 153, 90146 Palermo, Italy; antonino.scurria@ismn.cnr.it (A.S.); francesco.giordano@cnr.it (F.G.); mario.pagliaro@cnr.it (M.P.); 4 Istituto per la Bioeconomia, CNR, via Madonna del Piano 10, 50019 Sesto Fiorentino, Italy; francesco.meneguzzo@cnr.it; 5 Dipartimento di Scienze e Tecnologie Biologiche Chimiche e Farmaceutiche, Università di Palermo, Via Archirafi 32, 90123 Palermo, Italy; alessandro.attanzio@unipa.it; 6 Dipartimento di Biomedicina, Neuroscienze e Diagnostica Avanzata, Università di Palermo, Via Divisi 83, 90133 Palermo, Italy; stefania.raimondo@unipa.it

**Keywords:** pectin, cell cycle, hydrodynamic cavitation, oxidative stress, mitochondria, neurological disease, neurodegeneration, anticancer, antitumor, phytochemicals

## Abstract

Tested in vitro on SH-SY5Y neuroblastoma cells, grapefruit IntegroPectin is a powerful protective, antioxidant and antiproliferative agent. The strong antioxidant properties of this new citrus pectin, and its ability to preserve mitochondrial membrane potential and morphology, severely impaired in neurodegenerative disorders, make it an attractive therapeutic and preventive agent for the treatment of oxidative stress-associated brain disorders. Similarly, the ability of this pectic polymer rich in RG-I regions, as well as in naringin, linalool, linalool oxide and limonene adsorbed at the outer surface, to inhibit cell proliferation or even kill, at high doses, neoplastic cells may have opened up new therapeutic strategies in cancer research. In order to take full advantage of its vast therapeutic and preventive potential, detailed studies of the molecular mechanism involved in the antiproliferative and neuroprotective of this IntegroPectin are urgently needed.

## 1. Introduction

Globally contributing 16.5% of deaths from all causes and 11.6% of global disability-adjusted life-years in the year 2016, neurological disorders (NDs) are the second leading group cause of deaths in the world [1]. In recent years, NDs have increased significantly due to the ageing of the population, malnutrition, various forms of environmental pollution, lifestyle, diet [2], viral infections and other environmental and social factors [3]. Causing cell damage, oxidative stress is one of the main mechanisms involved in NDs, since it alters numerous cellular processes such as mitochondrial homeostasis [4], DNA repair and cell signaling, propagating cell damage and leading to incurable neurodegenerative diseases [5]. To date, no effective synthetic or natural drugs are available for preventing or treating NDs such as Alzheimer’s, Parkinson’s disease and amyotrophic lateral sclerosis. Hence, plentiful research has been devoted to search bioactive natural compounds to be used as neuroprotective and neuroregenerative agents. For example, algae-derived antioxidant molecules such as phycocyanins have been successfully used to inhibit cellular oxidative stress, mitochondrial dysfunction and apoptosis, and increase neuronal viability in an in vitro model of Alzheimer’s disease [6,7]. In general, antioxidant molecules from biological resources exhibit high bioavailability and higher efficacy than synthetic antioxidants [8]. A regular and correct intake of natural antioxidants plays an important role in the prevention and control of NDs, and several phytochemical complexes with antioxidant activity have been identified as potential therapeutic agents [9].

In this context, the ubiquitous plant heteropolysaccharide pectin is emerging as a “universal medicine” [10] thanks to its immunomodulating properties, including anti-inflammatory activity. The biopolymer is a natural component of all omnivorous diets and an important soluble dietary fiber promoting numerous beneficial effects on intestinal microbiota, preventing several inflammatory conditions [11]. Comprised of structurally distinct homogalacturonan (HG, accounting for 65%), rhamnogalacturonan (RG-I, accounting for 20–35%) and rhamnogalacturonan (RG-II, accounting for 2–10%) regions, pectin is a hydrocolloid abundant in the cell wall where it acts as glue, ensuring plant cell adhesion [12]. Owing to its excellent palatability, ability to provide texture at low concentration and excellent health and safety profile, pectin is the most valued natural hydrocolloid used by the food industry, with demand increasing at fast pace for more than a decade [13]. Furthermore, a number of new usages and applications of pectin have lately emerged that also contribute to its rapidly expanding demand [14]. The industrial production process is based on the hydrolysis of dried or wet citrus (lemon, orange and lime) peels (or apple pomace) with mineral acid in hot water followed by precipitation with isopropyl alcohol [15]. The process significantly degrades the structure of natural pectin, especially hydrolyzing the side chains of the RG-I “hairy” region [16]. The latter region is composed of a backbone of repeating galacturonic acid and rhamnose disaccharide with neutral sugar side chains attached to the *O*-4 position and sometimes the *O*-3 position of rhamnose backbone units. The neutral sugar side chains consisting of arabinose and galactose with variable linking types and chain lengths have demonstrated higher biological activity than the HG region of pectic polysaccharides [17].

From ultrasound-assisted and microwave-assisted extraction, numerous non-thermal and thermal methods to extract RG-I enriched pectins have been developed lately [16]. Among the latter, hydrodynamic cavitation has been lately applied to waste orange peel [18] as well as to the lemon and grapefruit [19] biowaste resulting from *Citrus* fruit juice extraction. Rich in flavonoids [20] and terpenes [21], the lemon “IntegroPectin” resulting from freeze-drying of the aqueous extract has an exceptionally high antioxidant activity and lacks cytotoxicity, even at a high concentration [19]. Furthermore, lemon and grapefruit IntegroPectin show significant antibacterial activity [22]. A subsequent detailed investigation has shown broad-scope antimicrobial activity for both newly obtained pectins, particularly for grapefruit IntegroPectin, which is a bactericidal agent at low concentrations against both Gram-positive and Gram-negative pathogenic bacteria [23].

Grapefruit IntegroPectin obtained upon freeze-drying is a low-methoxyl citrus pectin (degree of esterification, DE = 14% [23] compared to DE = 69% for commercial citrus pectin [24]), containing also a uniquely high amount of naringin (4′,5,7-trihydroxyflavonone-7-rhamnoglucoside), approaching 74 mg/g [20], adsorbed at its surface (for comparison, the highest yield values reported for naringin extracted from fresh grapefruit albedo is 10.5 mg/g [25]). Recently, we reported the powerful in vitro mitoprotective and neuroprotective activity of lemon IntegroPectin on neuronal SH-SY5Y human cells treated with concentrated (0.2 M) H_2_O_2_ [24]. Hydrogen peroxide is one of the key reactive oxygen species (ROS) involved in the cellular mechanisms leading to neurodegenerative pathologies whose overproduction in neurons leads to the oxidative degradation of neurons, rich in easily oxidized polyunsaturated fatty acids [26]. In this study, along with the high protective and antioxidant effect of grapefruit IntegroPectin on the same neuronal model cells, we report the discovery of powerful antiproliferative activity.

## 2. Results

### 2.1. Effect on SH-SY5Y Cell Viability

**Figure 1:**
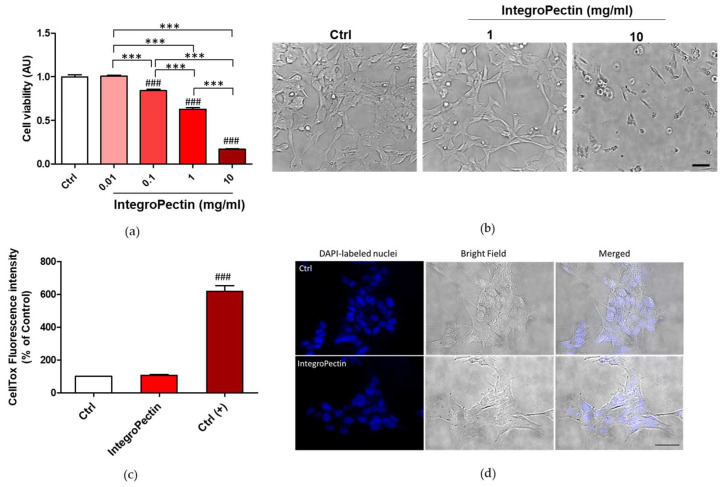
Dose dependent effects of grapefruit IntegroPectin on neuronal cell viability. (**a**) Cell viability of Integropectin treatment (24 h) in dose dependent experiment (*n* = 36); (**b**) Representative morphological images of untreated cells (Ctrl) or cells treated with different doses (1, 10 mg/mL) of IntegroPectin (24 h); (**c**) Cytotoxicity associated to IntegroPectin treatment (1 mg/mL, 24 h), evaluated by fluorescence developed using CellTox Green Cytotoxicity Assay (*n* = 13). Positive control Ctrl (+) is represented by cell exposure to lysis buffer; (**d**) Microscopy inspection of DAPI-labeled nuclei in Ctrl cells or treated with IntegroPectin (1 mg/mL, 24 h). Related bright field and merged pictures are also shown. Tukey test: ### *p* < 0.001 as compared to control (Ctrl) group; *** *p* < 0.001. Scale bar 50 µm.

We first evaluated the effect of different concentrations of grapefruit IntegroPectin on the cell viability of SH-SY5Y cells. Grapefruit IntegroPectin was solubilized as a powder in the cell culture medium. The powder dissolved quickly at room temperature requiring no heating, fast mechanical stirring or the employment of other solvents, including water or DMSO. As shown in Figure 1a, displaying the outcomes of the MTT (3-(4,5-dimethylthiazol-2-yl)-2,5-diphenyltetrazolium bromide) colorimetric assay, 24 h treatment with the new citrus pectin caused a dose-dependent reduction in cell viability, which became significant for doses exceeding 0.1 mg/mL. At 10 mg/mL dose, only a few cells remained viable. Similar results were obtained for treatment prolonged up to 48 h (Figure S1a), and in human lung carcinoma cells H292 treated for 24 h with grapefruit IntegroPectin 0.1–1 mg/mL (Figure S1b). However, while the dose of 10 mg/mL produced clear cytotoxic effects in SH-SY5Y cells, as evidenced by morphological analysis (Figure 1b), the 1 mg/mL dose was not associated to an alteration of cell morphology (Figure 1b) and to a significant increase in cell death, as shown by the cytotoxicity (CellTox) assay (Figure 1c) and by the healthy morphology of the cell nuclei counterstained with DAPI (4′,6-diamidino-2-phenylindole) fluorescent dye (Figure 1d).

Therefore, in order to understand the results of the MTT test and the absence of cell death at the dose of 1 mg/mL, we next analyzed the possible cytostatic effects.

### 2.2. Cytostatic Effect on SH-SY5Y Cells

**Figure 2:**
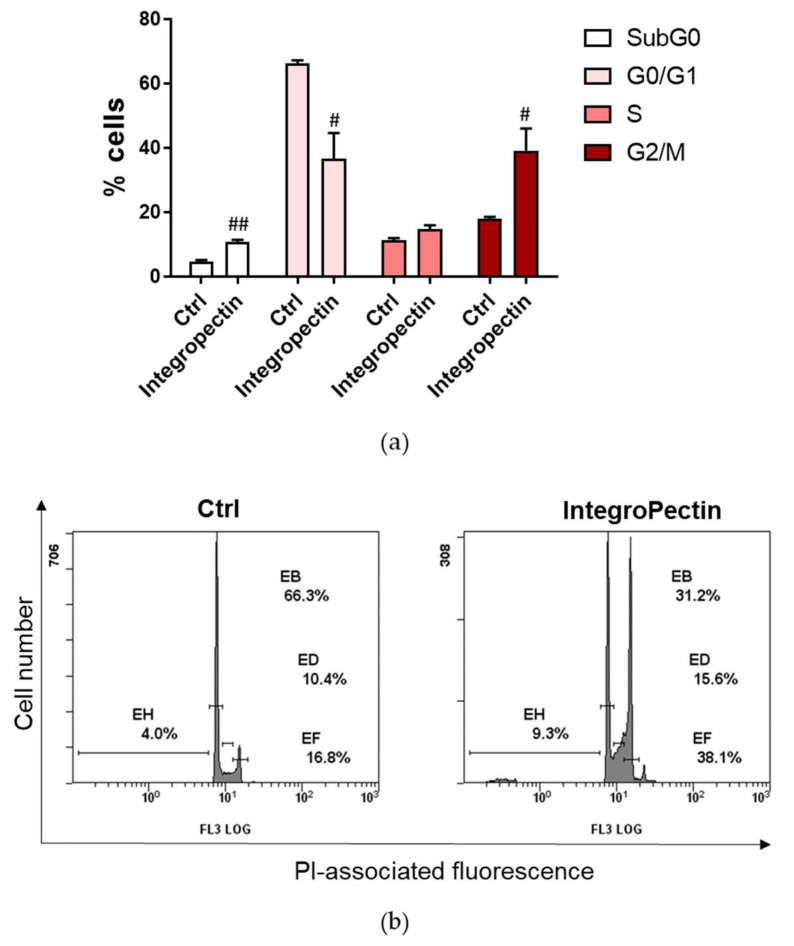
(**a**) Effect of grapefruit IntegroPectin on the cell cycle distribution of SH-SY-5Y cells. Percentage (%) of cell distribution of untreated (Ctrl) cells and cells treated for 24 h with IntegroPectin (1 mg/mL) in different phases of the cell cycle, assessed by flow cytometry analysis after propidium iodide (PI) staining (*n* = 12); (**b**) Representative images. *t*-test: # *p* < 0.05, ## *p* < 0.01 as compared to control (Ctrl) group.

The distribution of cells in the different phases of the cell cycle, analyzed by the flow cytometry analysis of cellular DNA content following cell staining with propidium iodide (PI), shed insight on the cytostatic effect of the new grapefruit pectin on neuronal model cells. As shown in Figure 2, treatment with grapefruit IntegroPectin produces a cell cycle arrest exactly at the G2/M phase. In the growth two phase (G2 phase) of the cell cycle preceding mitosis, the cell replenishes its energy and synthesizes the proteins needed for chromosome manipulation. Cell cycle arrest at the G2/M phase indicates that the damage of intracellular DNA is difficult to repair [27]. The G2/M-phase checkpoint usually prevents cells with damaged DNA from undergoing mitosis by the inhibition of the mitotic complex CDK1-cyclin B and activation of the apoptosis cascade [28]. However, we did not detect any increase in cell death when treatment was prolonged up to 5 days (Figure S2).

We thus assessed the potential antioxidant and protective properties of the new citrus pectin in cells exposed to concentrated (0.2 M) aqueous H_2_O_2_.

### 2.3. Protective Effect on SH-SY5Y Cells

**Figure 3:**
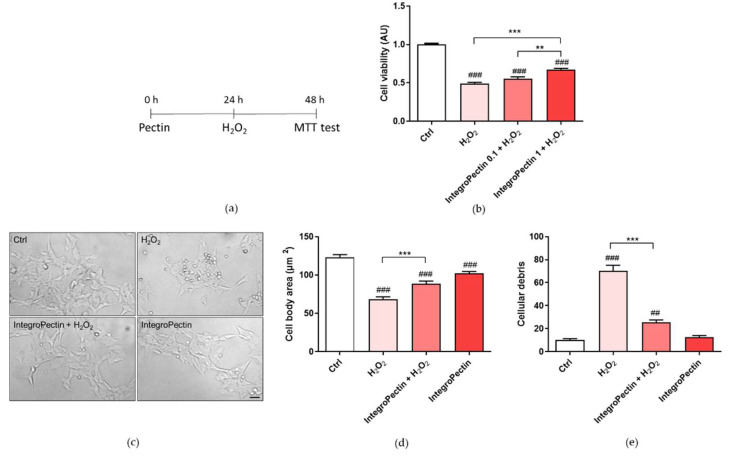
Effect of grapefruit IntegroPectin on cell viability and morphology of SH-SY5Y cells impaired by H_2_O_2_ treatment. (**a**) Scheme of cell pretreatment with IntegroPectin; (**b**) Histogram showing cell viability of untreated cells (Ctrl) or treated with IntegroPectin or with H_2_O_2_ alone or in combination with IntegroPectin (*n* = 45); (**c**) Representative bright field morphological images of untreated cells (Ctrl) or treated with IntegroPectin or with H_2_O_2_ alone or in combination with IntegroPectin; (**d**) Cell body area histogram of untreated cells (Ctrl) or treated with IntegroPectin or with H_2_O_2_ alone or in combination with IntegroPectin (*n* = 68); (**e**) Amount of cells debris per field histogram of untreated cells (Ctrl) or treated with IntegroPectin or with H_2_O_2_ alone or in combination with IntegroPectin (*n* = 36). Scale bar 50 μm. Tukey test: ## *p* < 0.01, ### *p* < 0.001 as compared to control (Ctrl) group; ** *p* < 0.01, *** *p* < 0.001.

Pretreatment (Figure 3a) with grapefruit IntegroPectin at a dose of 1 mg/mL, *per se* cytostatic, was able to significantly counteract the cell death induced by the treatment with 0.2 M H_2_O_2_ (Figure 3b). The same treatment was also able to recover cell morphology and cell body area, impaired by H_2_O_2_ treatment (Figure 3c,d). Furthermore, Figure 3c,e show that pretreatment with the newly sourced citrus pectin was able to reduce the amount of cell debris, indicative of cell protection. Similar results were obtained when the new grapefruit IntegroPectin was applied along with aqueous H_2_O_2_.

### 2.4. Antioxidative Effect on SH-SY5Y Cells

**Figure 4:**
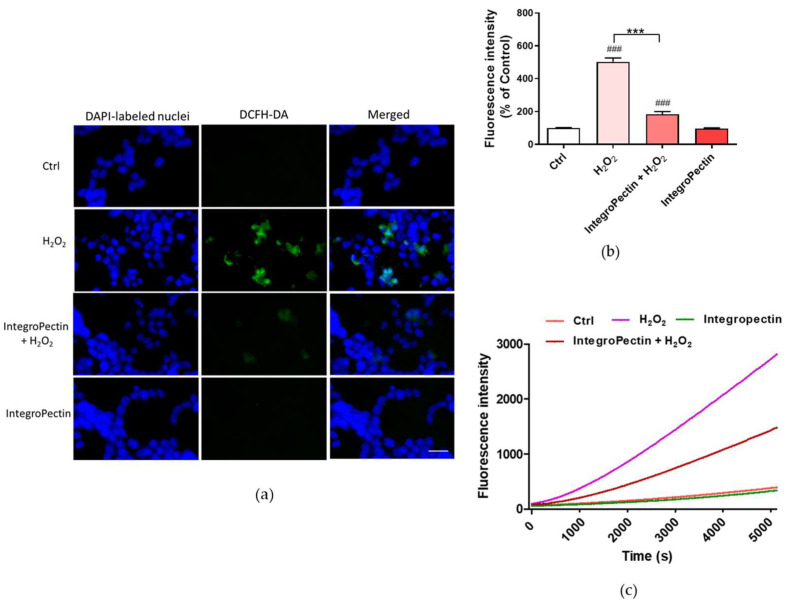
Effects of IntegroPectin on ROS production driven by exposure to aqueous H_2_O_2_. (**a**) DCFH-DA, DAPI and merged fluorescence microscopy images of untreated cells (Ctrl) or treated with In-tegroPectin or with H_2_O_2_ alone or in combination with IntegroPectin; (**b**) Histogram of fluores-cence intensity of untreated cells (Ctrl) or treated with IntegroPectin or with H_2_O_2_ alone or in combination with IntegroPectin measured using DCFH-DA fluorescence assay (*n* = 26); (**c**) Oxida-tion kinetics of untreated cells (Ctrl) or treated with IntegroPectin or with H_2_O_2_ alone or in com-bination with IntegroPectin, monitored using DCFH-DA fluorescence assay. Scale bar: 50 μm. Tukey test: ### *p* < 0.001 as compared to control (Ctrl) group, *** *p* < 0.001.

The impact of grapefruit IntegroPectin on H_2_O_2_-induced oxidative stress was assessed by ROS production using the dichlorofluorescein diacetate (DCFH-DA) fluorescence intensity assay, a widely used probe for detecting oxidative stress and intracellular reactive species [29]. A fluorescence microscope inspection (Figure 4a) and a fluorescence intensity measurement (Figure 4b) showed that the treatment of neuronal cells with grapefruit IntegroPectin almost completely counteracted the ROS formation driven by exposure to concentrated H_2_O_2_. The kinetics of ROS production after the exposure of SH-SY5Y cells to H_2_O_2_ shows that treatment with IntegroPectin is indeed highly effective in lowering and delaying ROS production due to hydrogen peroxide addition (Figure 4c).

### 2.5. Mitoprotective Effect on SH-SY5Y Cells

**Figure 5:**
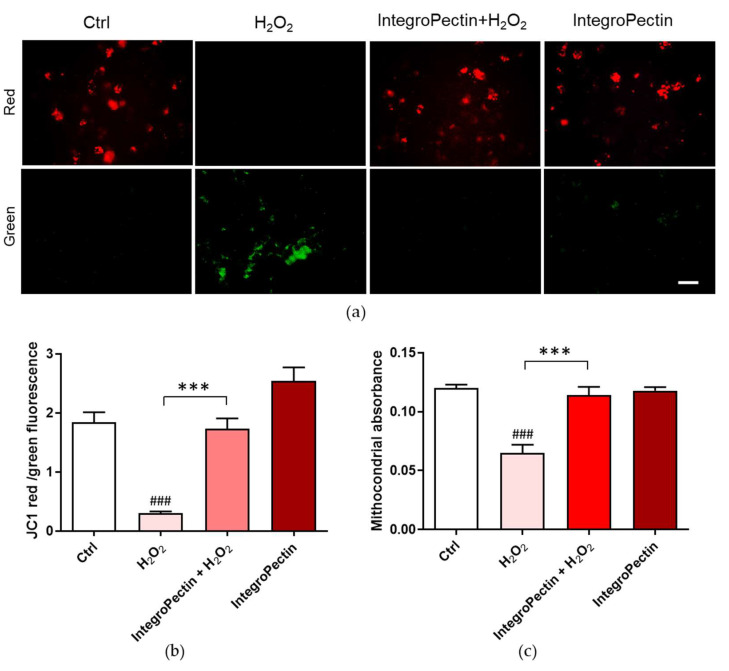
Effects of grapefruit IntegroPectin on mitochondria protection. (**a**) Fluorescence microscope inspection of untreated cells (Ctrl) or treated with IntegroPectin or with H_2_O_2_ alone or in combination with IntegroPectin submitted to JC-1 assay; (**b**) Histogram of the ratio between JC-1 red and green fluorescence intensity (*n* = 24); (**c**) Histogram of mitochondria absorbance (*n* = 24). Scale bar 100 µm. Tukey test: ### *p* < 0.001 as compared to control (Ctrl) group; *** *p* < 0.001.

Variations in the physiological mitochondrial membrane potential, an indicator of cells’ health and functional status in response to oxidative stress, were measured as changes in the accumulation of JC-1 cyanine dye red and green fluorescence signals in the cells. When excited at 488 nm, JC-1 monomers emit green fluorescence with a maximum at 530 nm (green), whereas J-aggregates emit orange-red fluorescence with a maximum at 595 nm (orange-red) [30]. The green fluorescent JC-1 dye forms red fluorescent aggregates when concentrated in energized mitochondria in response to their higher membrane potential, which is affected by oxidative stress. As displayed in Figure 5a,b, the JC-1 red/green fluorescent signal significantly decreased following cell exposure to H_2_O_2_, while the treatment of cells with IntegroPectin significantly reversed this effect.

Sustained mitochondrial damage triggers mitochondrial swelling due to increased colloidal osmotic pressure in the matrix accompanied by mitochondrial membrane depolarization and ATP hydrolysis. We assessed mitochondrial swelling by monitoring the decrease in light scattering at 540 nm. Treatment with the newly obtained IntegroPectin fully counteracted the significant H_2_O_2_-driven mitochondrial swelling (Figure 5c), supporting its mitoprotective function.

### 2.6. Structure of Grapefruit IntegroPectin

**Figure 6:**
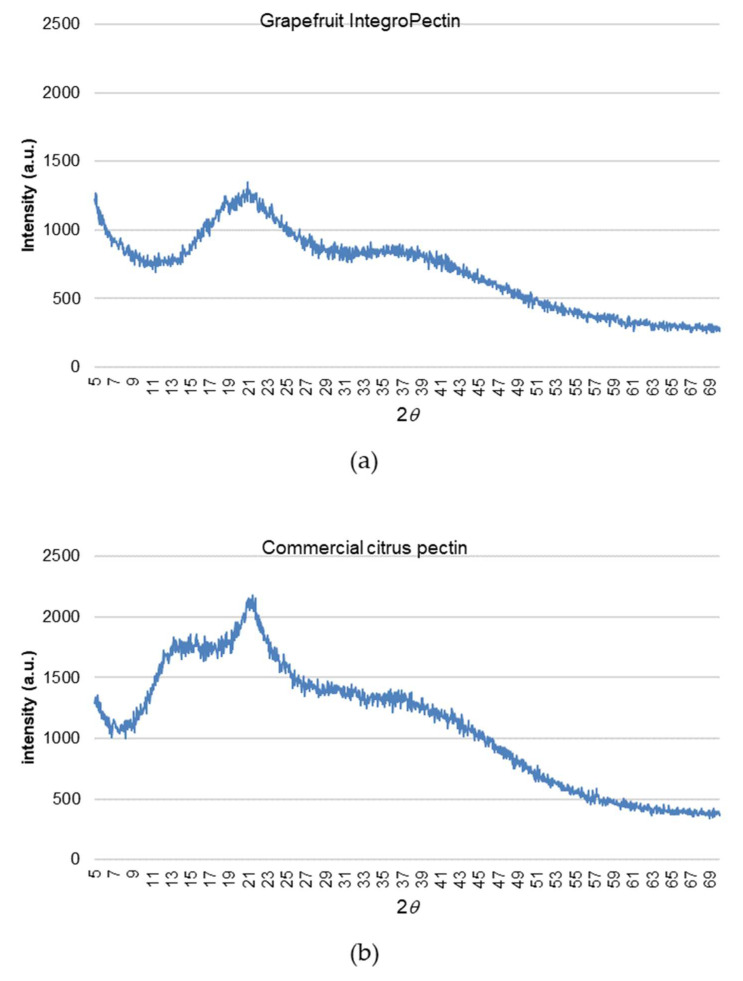
X-ray diffractograms of grapefruit IntegroPectin (**a**) and commercial citrus pectin (**b**).

From a structural viewpoint, grapefruit IntegroPectin is very different when compared to commercial citrus pectin extracted via conventional hydrolytic extraction in hot acidic water followed by precipitation with alcohol. Figure 6 shows the X-ray diffraction (XRD) patterns of grapefruit IntegroPectin and commercial citrus pectin.

Exactly as it happens with grapefruit pectin obtained via cavitation driven by ultrasounds, the diffraction peaks characteristic of the commercial citrus pectin at 12.4°, 14.3°, 21.0°, 28.9°, 31.5°, 32.2° and 40.2° [31] shift to higher 2*θ* values, with several sharp peaks disappearing [32]. This indicates that the hydrodynamic cavitation of grapefruit biowaste induces the nearly complete decrystallization of the homogalacturonan (HG) chains of the grapefruit pectin, following which it crystallizes in a hexagonal closest packing arrangement that ensures the closest packing of the chains (we remind readers that the diffraction from the HG regions is the only contributing to the XRD pattern) [33]. In further detail, these findings confirm that cavitation, no matter if acoustic or hydrodynamic, destroys the “fringed-micellar” structure of the crystalline regions of the semicrystalline pectin biopolymer [34]. This finding, along with the substantially higher number of RG-I regions and low DE, also explains the significantly larger solubility of the IntegroPectin in water at room temperature when compared to the poorly soluble commercial citrus pectin.

## 3. Discussion

Along with high in vitro protective and antioxidant action on the human neuroblastoma cell line SH-SY5Y, the grapefruit IntegroPectin extracted via hydrodynamic cavitation exerts powerful antiproliferative activity. On one hand, treatment with grapefruit IntegroPectin (10 mg/mL) produces cell death. On the other, a lower dose (1 mg/mL) is associated to cell cycle arrest exactly at the G2/M phase. Future studies will investigate whether the cell cycle arrest and antiproliferative effects observed might be indicative of cell differentiation process triggering. Undifferentiated SH-SY5Y cells, though sharing few properties with mature neurons, after differentiation to enhance their usefulness as neuronal models, even have an increased oxidative vulnerability [35]. In this respect, the high tolerance to oxidation of concentrated (0.2 M) H_2_O_2_, exhibited by the SH-SY5Y cells treated with 1 mg/mL of grapefruit IntegroPectin, along with the antiproliferative activity, is highly promising in light of future in vivo experiments hopefully leading to practical utilization of this new citrus pectin as an antioxidant and antitumor agent.

Due to its anti-cancer, anti-apoptotic, anti-atherogenic, anti-inflammatory and antioxidant properties, naringin, adsorbed at the grapefruit IntegroPectin surface at a nearly 74 mg/g load [20], is currently intensively researched in light of utilization for cancer prevention and treatment [36]. One of the main limitations to its use as a therapeutic agent is the poor solubility in water (0.5 g/L [37]), which leads to poor pharmacokinetics [36]. It is likely that its dispersion on the highly soluble fibers of grapefruit IntegroPectin obtained upon freeze drying enhances its bioavailability. Grapefruit IntegroPectin, furthermore, is rich in adsorbed limonene, linalool and linalool oxide (predominantly the *cis* isomer) [21]. Both linalool [38] and linalool oxide [39] have been lately shown to exert neuroprotective and anticonvulsant and antinociceptive activities in vitro and in vivo, respectively.

RG-I-enriched grapefruit IntegroPectin, however, does not act simply as a carrier of bioactive naringin, but possesses high bioactivity in itself [17], which magnifies the activity of the adsorbed flavonoid and terpenes. A similar finding was also observed for ultrasonically obtained grapefruit pectin, which, at 2 mg/mL concentration, has a free radical scavenging activity nearly twice higher than that of commercial citrus pectin [33]. The finding was ascribed by Liu and co-workers to its lower viscosity in solution, which would enhance the interaction between the pectin’s hydroxyl groups and free radicals [40]. It is also relevant to this study that enzymatically extracted apple pectin rich in rhamnose, fucose, protein and phenolic compounds was recently shown to possess antioxidant and antitumor activity on human adenocarcinoma and melanoma cell lines [41].

## 4. Materials and Methods

### 4.1. Solubilization of IntegroPectin

Grapefruit IntegroPectin was solubilized by dissolving 10 mg of pectic polymer powder in 1 mL of cell culture medium. The solution was filtered using a 0.45 µm sartorius filter and stored at +4 °C.

### 4.2. Cell Cultures and Treatment

SH-SY5Y cells were cultured in T25 tissue culture flasks in Complete Dulbecco’s Modified Grapefruit IntegroPectin was solubilized by dissolving 10 mg of pectic polymer powder in 1 mL of cell culture medium. The solution was filtered using a 0.45 µm sartorius filter and stored at +4 °C.

Eagle’s Medium and F12 (DMEM/F12; 1:1), supplemented with 10% fetal bovine serum (FBS), 100 U/mL penicillin and 100 U/mL streptomycin and 2 mM L-glutamine, in a humidified atmosphere of 95% air and 5% CO_2_ at 37 °C. The cell culture medium was changed every three days, and the cells were sub-cultured once they reached 90% confluence. The effects of grapefruit IntegroPectin were tested in cells cultured in 96-well plates. All treatments were performed at least 24 h after plating. Based on the experimental groups, the cells received the following treatments: H_2_O_2_ (200 µM for 24 h), Integropectin (10, 1, 0.1 and 0.01 mg/mL for 24 or 48 h), a combination of IntegroPectin and H_2_O_2_, with pectins administered 24 h before (pretreatment) or immediately before (co-treatment). The control (Ctrl) group was treated with an equal volume of cell medium.

### 4.3. Cell Viability and Cell Morphology

Cells were grown at a density of 2 × 10^4^ cell/well on 96-well plates in a final volume of 100 µL/well. Cell viability was assessed by measuring the reduction in a yellow tetrazolium salt (MTT, 0.5 mg/mL) to purple formazan crystals by mitochondrial succinate dehydrogenase expressed in metabolically active cells after 3 h incubation at 37 °C. Absorbance was measured at 570 nm with background subtraction after extracting MTT–formazan product with dimethyl sulfoxide (DMSO) 100 µL/well. Cell viability was expressed as arbitrary units, with the control group set to 1.

For the analysis of cell morphology, cells were grown at a density of 5 × 10^3^ cells/well on 96-well plates in a final volume of 100 µL/well. To this end, cells were fixed with 4% formaldehyde solution for 15 min at room temperature and washed twice with phosphate-buffered saline (PBS). For analysis of cell nuclei morphology, nuclei were counterstained with the fluorescent stain 4′,6-diamidino-2-phenylindole (DAPI). The cellular images obtained using the Zeiss Axio Scope 2 microscope (Carl Zeiss, Oberkochen, Germany) were analyzed with the ZEISS-ZEN imaging software, measuring each time the cell body size and the number of cell debris per field, and examining nuclei signal.

### 4.4. CellTox Green Cytotoxicity Assay

Cells were grown at a density of 2 × 10^4^ cell/well on 96-well plates in a final volume of 100 µL/well. Grapefruit Integropectin cytotoxicity was assessed using CellTox™ Green Cytotoxicity Assay (Promega Corporation, Madison, WI, USA). At the end of treatment, CellTox Green Reagent (100 µL) was added to each well. After 15 min of incubation, fluorescence was read using a Microplate Reader GloMax fluorimeter (Promega Corporation, Madison, WI, USA) at the excitation wavelength of 485 nm and emission wavelength of 530 nm. For positive control of toxicity, lysis solution was added to replicate wells 30 min before reading. After background subtraction, results were expressed as a percentage of the control group.

### 4.5. Flow Cytometry Analysis of Cell Cycle

Cells were grown at a density of 1.2 × 10^5^ cell/well on 24-well plates in a final volume of 500 µL/well. At the end of treatment, the cells were harvested by centrifugation, washed with PBS and incubated for 30 min in the dark in a PBS solution containing Triton X100 (0.1%), 20 μg/mL PI (Merck, Milan, Italy) and 200 μg/mL RNase (Thermo Fisher, Milan, Italy) according to a published method [42]. At least 1 × 10^4^ cells were analyzed for each sample.

### 4.6. Analysis of ROS and Oxidation Kinetics

To assess intracellular ROS concentration, SH-SY5Y cells were plated at a density of 1 × 10^4^ cells/well on 96-well plates in a final volume of 100 µL/well. At the end of the treatments, a 1 mM solution of 2′,7′-dichlorofluorescin diacetate (DCFH-DA, Merck, Darmstadt, Germany) dissolved in PBS was added to each well. The plate was thus placed in the dark for 10 min at room temperature for cell uptake. Upon cleavage of the acetate groups by intracellular esterases and oxidation, the nonfluorescent DCFH-DA is converted to highly fluorescent 2’,7’-dichlorofluorescein (DCF). The oxidation kinetics was investigated by placing SH-SY5Y cells at a density of 1 × 10^4^ cell/well on 96-well plates in a final volume of 100 µL/well. The kinetics of ROS production was evaluated for 90 min after the addition of H_2_O_2_. After washing with PBS, DCF fluorescence intensity was analyzed using the fluorescence Zeiss Axio Scope 2 microscope (Carl Zeiss, Oberkochen, Germany) along with a Microplate Reader GloMax fluorimeter (Promega Corporation, Madison, WI, USA) at the excitation wavelength of 475 nm and emission wavelength of 555 nm. Results were expressed as a percentage of the control group.

### 4.7. Mitochondrial Membrane Potential Analysis

The mitochondrial transmembrane potential was measured by incubating the cells for 30 min at 37 °C with 2 mM JC-1 red dye (5,5′,6,6′-tetrachloro-1,1′,3,3′-tetraethylbenzimidazolylcarbocyanine iodide) using the MitoProbe JC-1 assay kit (Molecular Probes, Eugene, OR, USA). Mitochondrial depolarization is indicated by a decrease in the red/green fluorescence intensity ratio, evaluated by the aforementioned fluorimeter and fluorescence microscope equipped with a 488 nm excitation laser.

### 4.8. Swelling of Isolated Mitochondria

The mitochondrial swelling was evaluated by measuring the decrease in the absorbance of the mitochondrial suspensions. The absorbance of isolated mitochondria was monitored for 5 min at 37 °C at 540 nm, using a GloMax Discover multimode plate reader (Promega Corporation, Madison, WI, USA).

### 4.9. XRD Measurements

The pectin samples were analysed using a D5005 X-ray diffractometer (Bruker AXS, Karlsruhe, Germany) operating at 40 kV and 30 mA. The X-ray radiation was generated via a copper (Kα) anode and made monochromatic via the instrument’s secondary monochromator. The diffraction profile of both grapefruit IntegroPectin and commercial citrus pectin (galacturonic acid ≥74.0 %, dried basis) purchased from Sigma-Aldrich (Merck Life Science, Milan, Italy) was acquired at 0.15°/min acquisition rate over the 5.0°–70.0° range.

### 4.10. Statistical Analysis

Data analysis was performed using GraphPad Prism 5 software (GraphPad Software, San Diego, CA, USA). The results are presented as mean ± SE, and in some cases are expressed as arbitrary units, with controls equal to 1, or as a percentage of control. Statistical evaluations were performed using one-way ANOVA, followed by Tukey’s Post hoc test, or *t*-test. Differences in *p*-value less than 0.05 were considered statistically significant.

## 5. Conclusions

In conclusion, tested in vitro on SH-SY5Y neuroblastoma cells, the grapefruit IntegroPectin rich in RG-I regions, as well as in naringin, linalool, linalool oxide and limonene adsorbed at the outer surface, exerts powerful protective, antioxidant and antiproliferative activities. The ability of this new pectin to inhibit cell proliferation or even kill, at high doses, neoplastic cells, coupled to the health-beneficial nature of pectin, citrus flavonoids and essential oils, may have opened up new therapeutic strategies in cancer research. In light of forthcoming in vivo and clinical trials of this newly developed citrus IntegroPectin, it is relevant that both pectin [43], citrus flavonoids [44] and citrus essential oils (terpenes and oxygenated compounds) [45] share an excellent health and safety profile.

Cancer is a multifactorial disease, involving both endogenous/exogenous factors, in which free radicals play a key role. ROS overproduction in cancer cells, particularly in the mitochondria [46], and the related accelerated oxidation of biomolecules facilitate mutagenesis, tumor growth and metastasis. The inhibition of the oxidative degradation process driven by ROS overproduction leads to benefits in tumor promotion and progression, especially considering the immunomodulating action of pectin and citrus flavonoids. Indeed, recent studies suggest the development of therapeutic strategies based on modulating ROS levels to treat cancer (ROS generated by different metabolic pathways also act as “Trojan horses” to eliminate cancer cells) [47]. Remarkably, epidemiological studies suggest that the incidence of cancer is lower in populations where the diet is rich in natural antioxidants such as those found in fruits and vegetables [48] Therefore, the combination of antioxidant, modulating and antiproliferative effects discovered in vitro for grapefruit IntegroPectin may represent a successful synergistic combination against tumor promotion and progression. Similarly, the strong antioxidant properties of grapefruit IntegroPectin and its ability to preserve mitochondrial membrane potential and morphology, severely impaired in neurodegenerative disorders, make this new biomolecule an attractive therapeutic agent for oxidative stress-associated brain disorders. Detailed molecular mechanism studies underlying the antiproliferative and neuroprotective effects of grapefruit IntegroPectin are urgently needed in order to take full advantage of its broad therapeutic and preventive potential.

## Data Availability

All experimental data are available by contacting the corresponding Authors.

## Supplementary Materials

The following are available online at https://www.mdpi.com/article/10.3390/ijms22179368/s1. Figure S1: Effects of grapefruit IntegroPectin on cell viability of SH-SY5Y and H292 cell lines. (a) Cell viability after IntegroPectin treatment (1 mg/mL) in time dependent experiment in SH-SY5Y cells (*n* = 33); (b) cell viability after IntegroPectin treatment (24 h) in dose dependent experiment in H292 cells (*n* = 18). Tukey test: ### *p* < 0.001 as compared to control (Ctrl) group; ** *p* < 0.01. Figure S2: Effects of long term (5 days) grapefruit IntegroPectin on cell viability of SH-SY5Y. (a) Cell viability after Integropectin treatment (5 days, 1 mg/mL) in SH-SY5Y cells (*n* = 12); (b) representative morphological images of untreated cells (Ctrl) or cells treated with IntegroPectin (5 days, 1 mg/mL); *T*-test: ### *p* < 0.001 as compared to control (Ctrl) group. Scale bar 50 μm.

## References

[1] (2019). GBD 2016 Neurology Collaborators. Global, regional, and national burden of neurological disorders, 1990-2016: A systematic analysis for the Global Burden of Disease Study 2016. Lancet Neurol..

[2] Picone P., Di Carlo M., Nuzzo D. (2020). Obesity and Alzheimer’s disease: Molecular bases. Eur. J. Neurosci..

[3] Nuzzo D., Picone P. (2020). Potential neurological effects of severe COVID-19 infection. Neurosci. Res..

[4] Picone P., Nuzzo D., Caruana L., Scafidi V., Di Carlo M. (2014). Mitochondrial dysfunction: Different routes to Alzheimer’s disease therapy. Oxid. Med. Cell Longev..

[5] Picone P., Nuzzo D., Giacomazza D., Di Carlo M. (2020). β-Amyloid peptide: The cell compartment multi-faceted interaction in Alzheimer’s disease. Neurotox. Res..

[6] Nuzzo D., Presti G., Picone P., Galizzi G., Gulotta E., Giuliano S., Mannino C., Gambino V., Scoglio S., Di Carlo M. (2018). Effects of Aphanizomenon flos-aquae (Klamin) extract on a cell model of neurodegeneration. Oxid. Med. Cell Longev..

[7] Nuzzo D., Contardi M., Kossyvaki D., Picone P., Cristaldi L., Galizzi G., Bosco G., Scoglio S., Athanassiou A., Di Carlo M. (2019). Heat-resistant Aphanizomenon flos-aquae (AFA) extract (Klamin) as a functional ingredient in food strategy for prevention of oxidative stress. Oxid. Med. Cell Longev..

[8] Kahl R., Kappus H. (1993). Toxicology of the synthetic antioxidants BHA and BHT in comparison with the natural antioxidant vitamin E. Z. Lebensm. Unters. Forsch..

[9] Nuzzo D. (2021). Role of Natural Antioxidants on Neuroprotection and Neuroinflammation. Antioxidants.

[10] Zaitseva O., Khudyakov A., Sergushkina M., Solomina O., Polezhaeva T. (2020). Pectins as a universal medicine. Fitoterapia.

[11] Beukema M., Faas M.M., de Vos P. (2020). The effects of different dietary fiber pectin structures on the gastrointestinal immune barrier: Impact via gut microbiota and direct effects on immune cells. Exp. Mol. Med..

[12] Daher Firas B., Braybrook S.A. (2015). How to let go: Pectin and plant cell adhesion. Front. Plant Sci..

[13] Seisun D., Zalesny N. (2021). Strides in food texture and hydrocolloids. Food Hydrocoll..

[14] Ciriminna R., Chavarría-Hernández N., Hernández A.R., Pagliaro M. (2015). Pectin: A new perspective from the biorefinery standpoint. Biofuel. Bioprod. Bioref..

[15] Ciriminna R., Fidalgo A., Delisi R., Ilharco L.M., Pagliaro M. (2016). Pectin production and global market. Agro Food Ind. Hi-Tech.

[16] Mao G., Wu D., Wei C., Tao W., Ye X., Linhardt R.J., Orfila C., Chen S. (2019). Reconsidering conventional and innovative methods for pectin extraction from fruit and vegetable waste: Targeting rhamnogalacturonan I. Trends Food Sci. Technol..

[17] Wu D., Zheng J., Mao G., Hu W., Ye X., Linhardt R.J., Chen S. (2020). Rethinking the impact of RG-I mainly from fruits and vegetables on dietary health. Crit. Rev. Food Sci. Nutr..

[18] Meneguzzo F., Brunetti C., Fidalgo A., Ciriminna R., Delisi R., Albanese L., Zabini F., Gori A., dos Santos Nascimento L.B., De Carlo A. (2019). Real-scale integral valorization of waste orange peel via hydrodynamic cavitation. Processes.

[19] Nuzzo D., Cristaldi L., Sciortino M., Albanese L., Scurria A., Zabini F., Lino C., Pagliaro M., Meneguzzo F., Di Carlo M. (2020). Exceptional antioxidant, non-cytotoxic activity of integral lemon pectin from hydrodynamic cavitation. ChemistrySelect.

[20] Scurria A., Sciortino M., Albanese L., Nuzzo D., Zabini F., Meneguzzo F., Alduina R.V., Presentato A., Pagliaro M., Avellone G. (2021). Flavonoids in lemon and grapefruit IntegroPectin. Preprints.

[21] Scurria A., Sciortino M., Presentato A., Lino C., Piacenza E., Albanese L., Zabini F., Meneguzzo F., Nuzzo D., Pagliaro M. (2021). Volatile compounds of lemon and grapefruit IntegroPectin. Molecules.

[22] Presentato A., Scurria A., Albanese L., Lino C., Sciortino M., Pagliaro M., Zabini F., Meneguzzo F., Alduina R., Nuzzo D. (2020). Superior antibacterial activity of integral lemon pectin from hydrodynamic cavitation. ChemistryOpen.

[23] Presentato A., Piacenza E., Scurria A., Albanese L., Zabini F., Meneguzzo F., Nuzzo D., Pagliaro M., Chillura Martino D., Alduina R. (2020). A new water-soluble bactericidal agent for the treatment of infections caused by Gram-positive and Gram-negative bacterial strains. Antibiotics.

[24] Nuzzo D., Picone P., Giardina C., Scordino M., Mudò G., Pagliaro M., Scurria A., Meneguzzo F., Ilharco L.M., Fidalgo A. (2021). New neuroprotective effect of lemon IntegroPectin on neuronal cellular model. Antioxidants.

[25] Victor M.M., David J.M., Sakukuma M.C.K., França E.L., Nunes A.V.J. (2018). A simple and efficient process for the extraction of naringin from grapefruit peel waste. Green Process. Synth..

[26] Collin F. (2019). Chemical basis of reactive oxygen species reactivity and involvement in neurodegenerative diseases. Int. J. Mol. Sci..

[27] Lezaja A., Altmeyer M. (2018). Inherited DNA lesions determine G1 duration in the next cell cycle. Cell Cycle.

[28] Schwartz G.K., Shah M.A. (2005). Targeting the cell cycle: A new approach to cancer therapy. J. Clin. Oncol..

[29] Kalyanaraman B., Darley-Usmar V., Davies K.J.A., Dennery P.A., Forman H.J., Grisham M.B., Mann G.E., Moore K., Roberts L.J., Ischiropoulos H. (2012). Measuring reactive oxygen and nitrogen species with fluorescent probes: Challenges and limitations. Free Radic. Biol. Med..

[30] Sivandzade F., Bhalerao A., Cucullo L. (2019). Analysis of the mitochondrial membrane potential using the cationic JC-1 dye as a sensitive fluorescent probe. Biol. Protoc..

[31] Sharma R., Kamboj S., Khurana R., Singh G., Rana V. (2015). Physicochemical and functional performance of pectin extracted by QbD approach from Tamarindus indica L. pulp. Carbohydr. Polym..

[32] Wang W., Ma X., Jiang P., Hu L., Zhi Z., Chen J., Ding T., Ye X., Liu D. (2016). Characterization of pectin from grapefruit peel: A comparison of ultrasound-assisted and conventional heating extractions. Food Hydrocoll..

[33] Palmer K.J., Hartzog M.B. (1945). An X-ray diffraction investigation of sodium pectate. J. Am. Chem. Soc..

[34] Gohil R.M. (2010). Synergistic blends of natural polymers, pectin and sodium alginate. J. Appl. Polym. Sci..

[35] Forster J.I., Köglsberger S., Trefois C., Boyd O., Baumuratov A.S., Buck L., Balling R., Antony P.M.A. (2016). Characterization of differentiated SH-SY5Y as neuronal screening model reveals increased oxidative vulnerability. J. Biomol. Screen..

[36] Ghanbari-Movahed M., Jackson G., Farzaei M.H., Bishayee A. (2021). A systematic review of the preventive and therapeutic effects of naringin against human malignancies. Front. Pharmacol..

[37] Pulley G.N. (1936). Solubility of naringin in water. Ind. Eng. Chem. Anal. Ed..

[38] Migheli R., Lostia G., Galleri G., Rocchitta G., Serra P.A., Bassareo V., Acquas E., Peana A.T. (2021). Neuroprotective effect of (R)-(-)-linalool on oxidative stress in PC12 cells. Phytomedicine Plus.

[39] Negromonte Souto-Maior F., Vilar da Fonsêca D., Rodrigues Salgado P.R., de Oliveira Monte L., Pergentino de Sousa D., Nóbrega de Almeida R. (2017). Antinociceptive and anticonvulsant effects of the monoterpene linalool oxide. Pharm. Biol..

[40] Ro J., Kim Y., Kim H., Jang S.B., Lee H.J., Chakma S., Jeong J.H., Lee J. (2013). Anti-oxidative activity of pectin and its stabilizing effect on retinyl palmitate. Korean J. Physiol. Pharmacol..

[41] Wikiera A., Grabacka M., Byczyński L., Stodolak B., Mika M. (2021). Enzymatically extracted apple pectin possesses antioxidant and antitumor activity. Molecules.

[42] Restivo I., Tesoriere L., Frazzitta A., Livrea M.A., Attanzio A., Allegra M. (2020). Anti-poliferative activity of a hydrophilic extract of manna from Fraxinus angustifolia Vahl through mitochondrial pathway-mediated apoptosis and cell cycle arrest in human colon cancer cells. Molecules.

[43] (2017). EFSA Panel on Food Additives and Nutrient Sources added to Food), Scientific Opinion on the re-evaluation of pectin (E 440i) and amidated pectin (E 440ii) as food additives. EFSA J..

[44] Ahmed O.M., AbouZid S.F., Ahmed N.A., Zaky M.Y., Liu H. (2021). An up-to-date review on citrus flavonoids: Chemistry and benefits in health and diseases. Curr. Pharm. Des..

[45] Bora H., Kamle M., Mahato D.K., Tiwari P., Kumar P. (2020). Citrus essential oils (CEOs) and their applications in food: An overview. Plants.

[46] Yang Y., Karakhanova S., Hartwig W., D’Haese J.G., Philippov P.P., Werner J., Bazhin A.V. (2016). Mitochondria and mitochondrial ROS in cancer: Novel targets for anticancer therapy. J. Cell Physiol..

[47] Perillo B., Di Donato M., Pezone A., Di Zazzo E., Giovannelli P., Galasso G., Castoria G., Migliaccio A. (2020). ROS in cancer therapy: The bright side of the moon. Exp. Mol. Med..

[48] Esquivel-Chirino C., Esquivel-Soto J., Morales-González J.A., Montes Sánchez D., Ventura-Gallegos J.L., Hernández-Mora L.E., Zentella-Dehesa A. (2013). Inflammatory Environmental, Oxidative Stress in Tumoral Progression. Oxidative Stress and Chronic Degenerative Diseases-A Role for Antioxidants.

