# Supplementary material for: Protective, Antioxidant and Antiproliferative Activity of Grapefruit IntegroPectin on SH-SY5Y Cells
Source: Int J Mol Sci. 2021 Aug 29;22(17):9368. doi: 10.3390/ijms22179368 (PMC8430642; doi:10.3390/ijms22179368)
Supplement: Supplementary file 1 [file ijms-22-09368-s001.zip › ijms-1336965-supplementary.pdf]

Protective, antioxidant and antiproliferative activity of grapefruit IntegroPectin on SH-SY5Y cells

Domenico Nuzzo, Miriana Scordino, Antonino Scurria, Costanza Giardina, Francesco Giordano, Francesco Meneguzzo, Giuseppa Mudò, Mario Pagliaro, Pasquale Picone, Alessandro Attanzio, Stefania Raimondo, Rosaria Ciriminna and Valentina Di Liberto

Supplementary Information

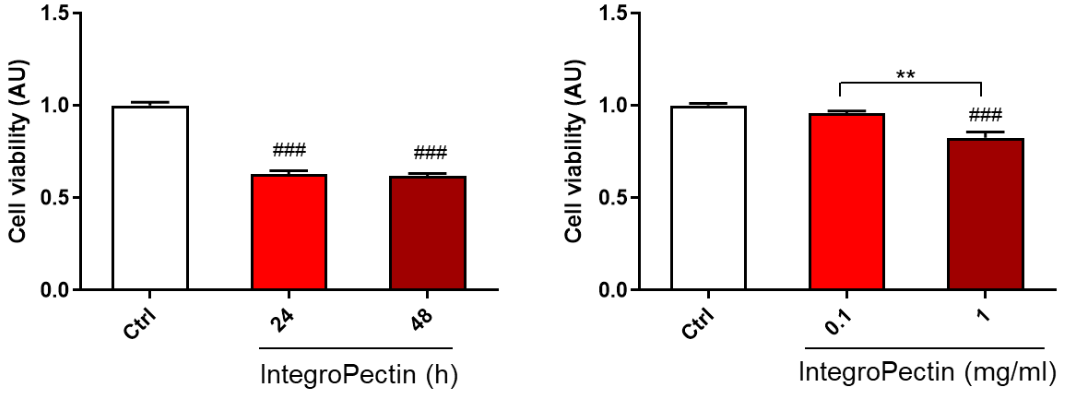

(a) (b)

Figure S1: Effects of grapefruit IntegroPectin on cell viability of SH-SY5Y and H292 cell lines. (a) Cell viability after IntegroPectin treatment (1 mg/ml) in time dependent experiment in SH-SY5Y cells (n=33); (b) cell viability after IntegroPectin treatment (24 h) in dose dependent experiment in H292 cells (n=18). Tukey test: ### $p < 0.001$ as compared to control (Ctrl) group; ** $p < 0.01$.

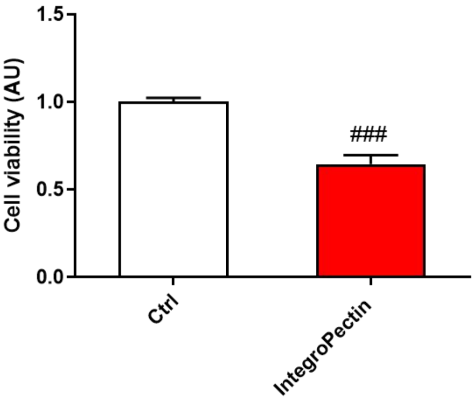

(a)

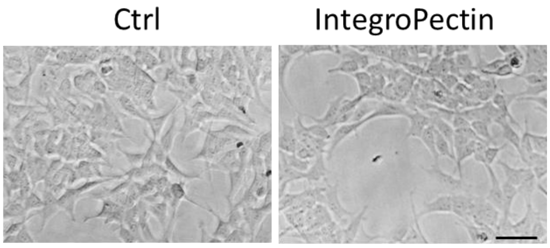

(b)

Figure S2: Effects of long term (5 days) grapefruit IntegroPectin on cell viability of SH-SY5Y. (a) Cell viability after Integropectin treatment (5 days, 1 mg/ml) in SH-SY5Y cells (n=12); (b) representative morphological images of untreated cells (Ctrl) or cells treated with IntegroPectin (5 days, 1 mg/ml); T-test: ### $p < 0.001$ as compared to control (Ctrl) group. Scale bar 50 μm.
